# Schroth Physiotherapeutic Scoliosis-Specific Exercise (PSSE) Trials—Systematic Review of Methods and Recommendations for Future Research

**DOI:** 10.3390/children10060954

**Published:** 2023-05-27

**Authors:** Sanja Schreiber, Daniel Whibley, Emily C Somers

**Affiliations:** 1 Department of Physical Therapy, Faculty of Rehabilitation Medicine, University of Alberta, Edmonton, AB T6G 2G4, Canada; 2 Curvy Spine—Specialized Scoliosis, Kyphosis and Other Spinal Disorders Centre, Edmonton, AB T6E 1W7, Canada; 3 Department of Physical Medicine and Rehabilitation, University of Michigan, Ann Arbor, MI 48109, USA; 4 Departments of Internal Medicine, Environmental Health Sciences and Obstetrics & Gynecology, University of Michigan, Ann Arbor, MI 48109, USA

**Keywords:** Schroth method, physiotherapeutic scoliosis-specific exercises (PSSE), exercise therapy, research design, data reporting, exercise trials

## Abstract

The Schroth method is a non-operative treatment for scoliosis and kyphosis, used standalone or as an adjunct to bracing. While supporting evidence for its effectiveness is emerging, methodologic standardization and rigor are equivocal. Thus, we aimed to systematically review methods of published Schroth physiotherapeutic scoliosis-specific exercise (PSSE) trials and provide guidance for future research. We searched six databases for randomized controlled trials (RCT) and non-randomized studies of interventions (NRSIs) investigating the effect of Schroth in children and adults with scoliosis or kyphosis. General characteristics, methodological approaches, treatment protocols, and outcomes reporting were analyzed. Risk of bias (RoB) was assessed using an adapted Cochrane RoB2 tool for RCTs and ROBINS-I for NRSI. Eligible studies (*n* = 7) were conducted in six countries and included patients with Scheuermann’s kyphosis (*n* = 1) and adolescent idiopathic scoliosis (*n* = 6). Though all seven studies used the term Schroth to describe their interventions, the Schroth method was used in four of seven studies, of which only one used Schroth classification, three used Schroth therapists, and none prospectively registered the study protocol. Overall, methodological rigor was suboptimal, potentially invalidating evidence synthesis. Authors should follow minimum standards for reporting, including prospectively registering detailed protocols; using appropriate exercise labeling, Schroth classification and certified therapists; naming and describing exercises per classification; and providing therapy dosages, prescription methods, and adherence.

## 1. Introduction 

Scoliosis is an abnormal curvature of the spine that displaces the spine in all three planes of the body away from its natural position, creating morphological changes in the trunk and intervertebral discs, leading to structural changes in the vertebrae that can develop at any stage of life. Adolescent idiopathic scoliosis (AIS) has an unknown etiology [1,2,3,4], and is the most common type of scoliosis, accounting for 90% of scoliosis cases [5]. AIS is progressive, with onset typically during puberty in a healthy child [1] affecting 0.47–5.2% of the population [5]. Females are more affected, and as curve severity increases, the female to male ratio increases up to 10:1 [1,6,7]. Progression is associated with pain, limitation in function, external deformities, and reduced self-image, pulmonary function, and health-related quality of life (HRQL) [1,8,9,10,11,12]. Skeletally immature patients have the highest risk of progression during the pubertal growth spurt [13], and early treatment is paramount.

Adult spinal deformity (ASD) or adult scoliosis is a complex spinal disorder that can broadly be categorized into a continuation of childhood scoliosis and degenerative (*de novo*) scoliosis with an onset in adulthood [14]. Degenerative changes may also affect pre-existing childhood scoliosis, which could lead to a progression and decreased HRQL [14,15,16]. The prevalence of adult scoliosis in those above 18 years of age is estimated at 11.3%, and at 68% in those over the age of 70 [17]. If symptomatic, ASD profoundly affects HRQL, including debilitating pain, disability, and neurological problems [17,18,19,20]. Several classifications of ASD have been developed based on etiology as well as correlation between radiological and HRQL outcomes [14,15,16,21]. Biomechanically, ASD presents differently than adolescent idiopathic scoliosis, and it has a higher rate of progression [17]. Appropriate clinical and radiological assessment, as well as the risk of progression estimation of individuals with ASD is paramount to ensure the optimal therapeutic intervention which should involve multidisciplinary teamwork.

Contrary to scoliosis, hyperkyphosis is a deviation of the normal physiological spinal alignment in the sagittal plane. Scheuermann’s kyphosis, also known as Scheuermann’s disease, is a special type of hyperkyphosis in which the thoracic or thoracolumbar spine becomes very rigid and fixed in excessive kyphosis due to the vertebral bodies being wedged. Diagnosis is made based on lateral radiographs and is established if the thoracic or thoracolumbar kyphosis measure >40° or <30°, respectively, and if at least three adjacent vertebrae demonstrate wedging of ≥5° [22,23]. Similar to scoliosis, the etiology is not well understood, and conversely, it is slightly more male predominant, with a roughly 2:1 male to female ratio and affecting approximately 5% of population [22,23]. The onset of the disease is typically between 12 and 17 years of age. Natural history of Scheuermann’s disease demonstrates that the condition may lead to back pain, disability, cardiopulmonary issues, psychological distress due to physical appearance, and decreased function [22,23,24]. The curves progress with longitudinal growth, and early treatment is essential.

The management of AIS and Scheuermann’s kyphosis is limited by our lack of understanding of their etiology and pathogenesis. To determine the etiology of scoliosis, some authors have suggested that a generalized growth or functional defect within the skeletal muscle could be one of the causes [2]. Evidence suggests that the distribution of the Type I muscle fibers, responsible for postural control, of the erector spinae on the convex side is significantly higher than that on the concave [25,26]. In addition, an extensive literature review on the etiology of AIS reported that children with AIS show abnormalities in postural balance, somatosensory function equilibrium, and proprioceptive function [27]. Moreover, because children with AIS grow faster than typically developing children, it is postulated that postural mechanisms of the somatic nervous system are incapable of controlling the initiating deformity [27,28]. Due to these anatomical and physiological imbalances, the length–force relationships can be altered in postural muscles due to muscle adaptation [29]. The asymmetrical loadings on the spine can cause further progression during skeletal growth in a perpetual “vicious cycle” unless some counter force halts it [30,31,32,33,34]. Stokes *et al*. conducted a biomechanical study on the progression of scoliosis and found that curve progression depends on an individual’s neuromuscular control of the trunk muscles, *i.e.*, muscle activation strategy [33,35]. The authors concluded that to reverse the “vicious cycle”, auto-correction must be at the maximum level [35]. Auto-correction is defined as the ability to reduce the spinal deformity through the patient’s active postural realignment of the spine in three dimensions [36].

Thus, it can be concluded that the effective exercise treatment for scoliosis and hyperkyphosis in children must (a) be introduced in the early stages of scoliosis development and applied throughout the growing phase of adolescence [37,38,39]; (b) address auto-correction in all three dimensions [40,41,42,43,44]; and (c) be applied in the direction opposite to the curve pattern under loading for long periods of time [30,31,45]. In adults, treatment of spinal deformity targets symptom management, including pain, HRQL, and disability. Operative and non-operative scoliosis-specific treatment for adult spine deformity target decompression and stabilization of the spine [46,47].

The Schroth method is a physiotherapeutic scoliosis-specific exercise (PSSE) approach consisting of neuromuscular, postural, and breathing exercises and education on adjusted activities of daily living. The Schroth method capitalizes on auto-correction and principles of motor learning and control, and is highly individualized [48,49]. This PSSE approach uses a specific Schroth classification system to categorize patients with scoliosis into 16, and those with sagittal plane deformity into 3 curve classifications [48,49]. The clinical and radiological assessment starts with the Schroth classification, which determines treatment prescription. The main goal of the Schroth treatment is recalibration of normal postural alignment and static/dynamic postural control, as well as improving spinal stability [48,49].

Since 2015, when the first trials on the Schroth method were published [50,51], the method has attracted interest among researchers, clinicians, patients, and their families. Despite increasing popularity, there is a lack of understanding of what constitutes the Schroth method, its effects, and in which populations it should be used. A growing body of primary studies on the Schroth method lacks methodological rigor, thus making collation of the evidence into systematic reviews and meta-analyses challenging and potentially invalid.

Therefore, we aimed to systematically review methods of published Schroth PSSE controlled trials for scoliosis and kyphosis and provide guidance for future research.

## 2. Materials and Methods

We included randomized (RCT) and non-randomized studies of intervention (NRSIs) that investigated the effects of Schroth in pediatric and adult populations with scoliosis or kyphosis. We excluded observational studies, uncontrolled studies or case series, reviews, secondary studies, and trials that did not investigate the Schroth treatment or that used Schroth as a control.

We searched six databases for the term “Schroth” (PubMed, Embase, Scopus, CINAHL, SPORTDiscus, and PEDro) from inception to 24 January 2022. The full search strategy for all databases is provided in Supplementary File S1. We augmented our search by reviewing published abstracts, as well as the US National Institutes of Health Trials Registry platform http://clinicaltrials.gov during the review period to identify published protocols of completed, unpublished, and ongoing trials.

To streamline the production of our review, we used Covidence, a web-based systematic review collaboration software platform (Veritas Health Innovation, Melbourne, Australia; available at www.covidence.org). We conducted all steps of the review, including abstract and title review, full text review, risk of bias assessment, and data extraction in Covidence.

Two independent reviewers screened titles/abstracts and full texts based on predefined criteria. In case of conflicts, the reviewers discussed until consensus was reached.

General characteristics (9 domains), methodological approaches (14 domains), treatment protocols (12 domains), and outcomes reporting (20 domains) were extracted into 55 domains and compared. The list of domains extracted for this review is provided in Supplementary File S2.

Risk of bias (RoB) was assessed using an adapted Cochrane RoB 2 tool for RCTs [52], and the Risk of Bias In Non-Randomized Studies–of Interventions (ROBINS-I) for NRSIs [53]. To assess the rigor of the trials more precisely, instead of using seven predetermined risk of bias domains for RoB 2, we augmented the domains to include: Sequence generation, Allocation concealment, Blinding of participants, Blinding of the study therapists, Blinding of outcome assessment—evaluator, Blinding of outcome assessment—statistician, Incomplete outcome data, Attrition, Selective reporting, Baseline group characteristics, Cointervention, Compliance, Outcome detection, Other sources of bias, and Overall as per Cochrane Back and Neck group recommendations [54]. For ROBINS-I, we used the following risk of bias domains: Bias due to confounding, Bias due to selection of participants, Bias in classification of interventions, Bias due to deviations from intended interventions, Bias due to missing data, Bias in measurement of outcomes, Bias in selection of the reported result, and Overall. The description of domains for each risk of bias tool and signaling questions that guided our decision making related to quality assessment are presented in Supplementary File S3. We used the Risk-of-bias VISualization (robvis) tool to create risk-of-bias plots and summary tables [55].

In surgical trials, the length of a follow-up is considered adequate if at least two years duration [54], while consensus between two leading societies on non-operative and operative management of scoliosis, the international Society on Scoliosis Orthopaedic Rehabilitation Treatment (sosort.org) and Scoliosis Research Society (srs.org), respectively, recommends a minimum of one-year follow-up for therapeutic trials [56]. Moreover, many journals that publish articles on spinal deformities do not accept submissions of manuscripts without at least 2-year follow-up on all patients. Surgery and physiotherapy have different goals, and the nature of these treatments is substantially different. Therefore, the outcome time points should be interpreted in a clinically meaningful way. In our review, we adopted recommendations of the Cochrane Back and Neck group for exercise trials and classified studies into *short-term* (≤4 weeks), *intermediate* (between 4 weeks and 1 year), and *long-term* (≥1 year) [54].

We verified whether a study used Schroth-certified therapists through publicly available registers. If a treating therapist could not be verified, we directly contacted official instructors and the authors.

## 3. Results

### 3.1. General Characteristics, Methodological Approaches, Treatment Protocols, and Outcomes Reporting

#### 3.1.1. General Characteristics of the Included Studies

Aside from identifying study information (lead author name, affiliation, contact, study I.D., and title), we also extracted information on the country where the study was conducted, setting, funding sources, and possible conflicts of interests for study authors. Detailed information is available in Supplementary File S2.

Two studies were conducted in Turkey, and the others in Canada, Israel, Saudi Arabia, the Republic of Korea, and the USA. Six studies were conducted in outpatient hospital clinics, and for one the setting was not reported. Two studies received no funding, four studies did not mention sources of funding, and one study received funding from multiple non-profit and foundation sources. We did not identify any potential conflicts of interests in any of the included trials.

#### 3.1.2. Methodological Approaches of the Included Studies

To describe methodological aspects of the included studies, we extracted information on the goal of the study, design, population, curve types, inclusion and exclusion criteria, method of recruitment, protocol registration, usage of specific Schroth classification, bracing information, primary and secondary outcomes, and information on questionnaires used. Detailed information is available in Supplementary File S2.

Of the included studies, six were randomized and one was a non-randomized study described by the authors as a prospective cohort study.

Four studies did not register protocols for their studies, two registered after the completion of the study, and one registered after the start but before the end of the study.

Six studies assessed the effect of the intervention versus a control treatment, while one assessed the effect of the supervised versus non-supervised and no treatment intervention. The control treatment varied among studies, including standard of care in two, followed by proprioceptive neuromuscular facilitation techniques (PNF), Pilates, antigravitation exercises, core exercises, and no treatment. The standard of care in one study included observation (no active treatment) and in the other observation or bracing depending on the standard of care indication.

All studies included pediatric patients: two studies included adolescents aged from 10 to 17, three from 10 to 18, one from 14 to 16 years, and one did not specify *a priori* inclusion by age, but the results suggest an average age of 15 to 16 years. Six studies focused on AIS and one on Scheuermann’s kyphosis. Two studies included participants with smaller curves ≤25°, three studies included curves from 10° to 30° and 10° to 45°, one from 10° to 60°, and one did not specify the inclusion based on the Cobb angles *a priori*.

Exclusion criteria varied. Most common exclusions were scoliosis type other than AIS, previous spine surgery, comorbidities, mental or neurological deficits, and inability to commit to the protocol. Six studies excluded patients who were currently or previously braced, and one study included patients with a brace if it was indicated and prescribed but excluded those who had completed brace treatment.

In six studies, patients were recruited based on their diagnosis from hospital out-patient clinics. One study did not specify how the patients were recruited.

Three scoliosis studies reported including specific curve types ranging from all curve types to only thoracolumbar; one included thoracic, double major, and thoracolumbar/lumbar, while others did not report on curve types. Only one study reported specifically using a Schroth classification and stratification according to the Schroth curve type.

The Cobb angle was the primary outcome in all included studies. Secondary outcomes included objective measures of incidence of progression, brace prescription, angle of trunk rotation, static plantar pressure distribution, functional capacity, muscle endurance, waist asymmetry, weight distribution, surface topography measurements, spinal mobility, and subjective measures on pain, body image perception, and quality of life. Patient-reported outcomes included the Spinal Appearance Questionnaire, the Scoliosis Research Society SRS-22r (three studies), and the SRS-23 (one study) questionnaires as well as the numeric pain rating scale (NPRS). The study including participants with kyphosis included two kyphosis-specific clinical outcomes: kyphotic deformity measured using inclinometer and introduced a new outcome that the group named L5 kyphosis apex line (L5-KAL) which has not been assessed for validity and reliability.

#### 3.1.3. Treatment Protocols of the Included Studies

Treatment protocol information included titles of the Schroth exercises, number and whether Schroth-certified treating therapists were used, description of the intervention and control, start and end date, duration of the study period, intervention intensity, intensity progression, adherence monitoring and calculation, as well as reporting on therapy performance. Detailed information is available in Supplementary File S2.

Of seven included studies, six reported using Schroth exercises, of which four indeed corresponded to the Schroth method, one to the Barcelona Scoliosis Physical Therapy School (BSPTS) approach, and for one it was unclear what approach was used. One of the studies reported to have used Schroth-based exercises that corresponded to the BSPTS approach. Labeling of the exercises was performed in five studies, while appropriate description was provided in three studies. For the remaining two studies that included the names of the exercises, for one the exercise did not correspond to the Schroth method and for the other, while the names of the exercises were correct, the descriptions were not. It was unclear how many therapists were involved in the treatment for four studies, two reported to have used one, and one study two therapists. Authors of the studies were included in the Schroth (four studies) and BSPTS registrars (two studies). In one study, the authors or the study personnel could not be verified as Schroth trained. Four studies explicitly stated to have used either Schroth or BSPTS therapists.

Trial durations were intermediate to long-term, including 10 weeks in one study, 12 weeks in another, 6 months in three studies, and 1-year follow-up in two studies.

Protocol adherence was reportedly monitored in three studies, with only two quantifying the adherence, but only one explicitly describing how adherence was calculated. A single study explicitly monitored exercise performance using a predefined study performance checklist, while one reported to have used a therapist’s non-specified subjective observation. The exercises were progressed in two trials. One study used timed progression in three phases: early phase during the first 3 weeks, mid-phase from the 4th to 7th week, and advanced phase occurring during the last 4 weeks. The other study used the progression algorithms designed for each curve type, guiding the progression from easier to more challenging exercises, and from start to target intensity, including less and more sets and reps, respectively.

#### 3.1.4. Outcomes Reporting of the Included Studies

Outcomes reporting included number of participants, baseline characteristics, age, sex, race/ethnicity, Risser stage, scoliosis classification, and Cobb angles.

All studies included a relatively small sample, ranging from 24 to 50 participants. Two studies included only females. Only one study specifically reported on race/ethnicity.

All but one study reported on the Risser stage. One included only Risser 0, two all Risser stages, one Risser 0 to 3, one Risser 1 to 4, and one Risser 2 to 4.

The mean Cobb angle in the studies including participants with scoliosis ranged from 16° to 33.4°, with four studies including patients below the bracing threshold.

Two studies did not report on any type of curve classifications. Two studies included only one curve type: one major lumbar and one major thoracic. Other studies did not limit inclusion by the curve type. Only one study reported to have used the Schroth classification.

The table in Supplementary File S4 summarizes variables reported across the studies.

### 3.2. Risk of Bias Assessment

#### 3.2.1. Risk of Bias of Included RCTs

Sequence generation was adequately reported for the most part, except for two studies. For one study, there was a lack of information regarding envelope concealment, as well as not mentioning who dealt with the envelopes; thus, the study was assessed with “some concerns”. Another study stated that participants were randomized without providing any details regarding how randomization was conducted, and was thus labeled “high risk” for this domain.

Allocation concealment was adequate in three studies. Two studies were considered to have “some concerns” due to a lack of information regarding envelope concealment and the difficulty to appraise whether allocation could have been foreseen in advance or during enrolment. Another study did not provide details regarding whether the allocation sequence was generated and, if it was, how it was conducted, and was therefore assessed as having a “high” risk of bias for this domain.

Blinding of participants and study therapists is not possible in physiotherapy trials due to the nature of treatment delivery. Therefore, all included studies were assessed as “high risk”. However, we considered detection bias (blinding of assessors and statistician) a greater potential contributor to the systematic error in results of these trials. Thus, we did not penalize the trials based solely on the blinding of participant and therapist domains.

Blinding of outcome assessment—evaluator and statistician is critical for avoiding detection bias. All but two RCTs reported on blinding evaluators, and only one reported on blinding the study statistician. Thus, two studies were considered to have “some concerns” regarding the blinding of outcome assessors, and five regarding blinding of the study statistician.

To assess the Incomplete outcome data domain, we considered whether the patients were analyzed as randomized, and whether missing data were discussed and accounted for regardless of participants’ reported adherence and potential cointervention. Two studies were assessed as having “high risk” of bias because one did not use intention-to-treat analysis and reported a 12% dropout rate, and another did not include a CONSORT diagram, which made it difficult to assess if the sample size reflected everyone who was recruited or if this represented participants with complete data. Other studies were labeled as having a “low risk” of bias on this domain, with three reporting no data loss, and one undertaking an intention-to-treat analysis.

Attrition was assessed with regard to trial duration and the reporting of the reasons for drop-out. In a short-term study, a drop-out of >20% and in a long-term study, a drop-out of >30% were considered unacceptable. Three studies reported no dropouts, two reported a 12% dropout rate in an intermediate 6-month- and 1-year-long study, thus receiving a “low” risk of bias for this domain. The remaining study was labeled as “high risk”, because it did not report on the number of participants who were approached and who were finally recruited in the study.

Selective reporting was “high risk” in two studies for which a protocol was not available and the verification of planned compared to reported outcomes was impossible. In addition, one of these studies mentioned pain as an outcome in the discussion but did not report on that in the results section; the other excluded two participants from the intervention group due to having “extremely high values of curves”, suggesting purposeful selection of reports. Another study was assessed with “some concerns” due to the lack of a protocol.

Baseline group characteristics were assessed as “high risk” in one study in which age, height, and curve magnitude measured by inclinometer were different between the groups. In addition, in the same study, the intervention group consisted primarily of boys, while control consisted primarily of girls (64% vs. 20%, respectively).

Cointervention was considered as having “some concerns” for four studies because cointervention and whether the authors controlled for possible cointervention were not mentioned. The other two RCTs specifically addressed cointervention, and this domain was assessed as “low risk” for these trials.

Compliance was adequately reported in one RCT, reporting on how the compliance was monitored and calculated, including the report of adherence in both intention-to-treat and per-protocol analyses. Two studies did not mention adherence and were scored with a “high risk” of bias on this domain, while the remaining three RCTs were assessed as having “some concerns” in this domain due to either not reporting on adherence or not reporting on how the adherence was monitored.

Outcome detection or the timing of when the outcomes were measured was adequately reported (“low risk”) in all but one RCT, for which it was unclear whether the outcomes were measured concurrently in both groups, thus scoring “some concerns” on this domain.

Other sources of bias—This is a “catch-all” domain where additional potential sources of bias could be listed. Additional sources of bias were found in three studies. Most commonly, a source of bias included a lack of published protocol or a protocol that was published after the study was completed, which was found for four studies. Two studies published protocols after the completion of the trials, with one having an ethics approval available and comparable to the protocol, while the protocol for the other study was judged to be very poor and lacking detail, and was therefore rated as having a high risk of bias. One study was conducted in Saudi Arabia, while both authors’ affiliations were at an institution in Egypt, and the protocol was published in a Japanese registry and lacking important information. We considered that a high risk of bias. For the same study, it was not possible to verify whether qualified therapists were used, in addition to the exercise descriptions not corresponding to the treatment they reported to have used, signaling a possible incorrect use of the treatment. We considered that to be high risk of bias under this domain.

Overall—We considered overall risk of bias as “high” or “some concerns” if at least one domain was assessed as “high” or “some concerns”, except for the blinding of therapist or participant domains. “Low” risk of bias was assigned to studies that had all but blinding of therapists and participants domains judged as “low”. Therefore, three RCTs were considered as “high”, two with “some concerns”, and one as “low” risk of bias.

**Figure 1:**
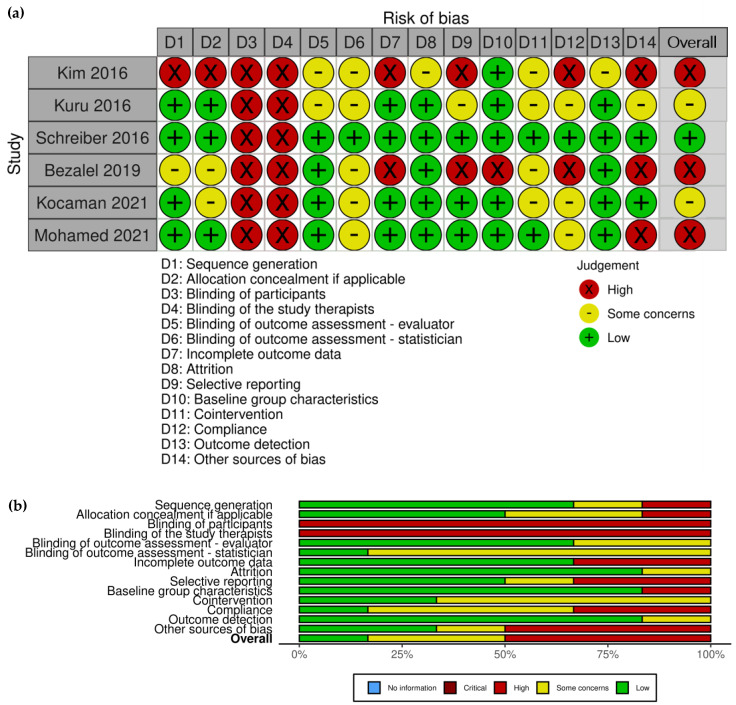
(**a**) Modified RoB 2 of included randomized controlled trials (RCTs) with corresponding domains; (**b**) Risk of bias summary plot.

The summary plot corresponding to the Modified RoB 2 for included RCTs is presented in Figure 1.

#### 3.2.2. Risk of Bias in Non-Randomized Studies (ROBINS) of Included NRSIs

Bias due to confounding is an important aspect in designing NRSIs because some prognostic variables could also predict the intervention received at baseline. For the included NRSI study, this domain was assessed as “moderate risk” due to baseline characteristic differences, including a discrepancy in sex and curve type between the groups that the authors did not control for in the analysis.

Bias due to the selection of participants is another critical domain in NRSI conduct, because exclusion of some eligible participants at the beginning of the trial may lead to a systematic error. For the included NRSI, it was not possible to ascertain how the participants were selected at baseline, if enrollment was consecutive or based on convenience, or if the participants were naïve to the treatment, and this domain was scored as “serious” risk.

Bias in the classification of interventions and bias due to deviations from intended interventions were considered as “low risk” because both intervention and control treatment are standard of care at the study institution, and it was assumed that both would be carried out in their usual ways.

Bias due to missing data corresponded closely to the “Attrition” domain for RCTs. The included NRSI reported a dropout rate of 32.7%, which is considered unacceptable for a long-term study, and received a “Critical” score.

Bias in the measurement of outcomes closely corresponded to Outcome detection in RCTs. The included NRSI scored “Moderate” risk because it was unclear what the actual timing was compared with baseline visits since it varied for each participant and could be every three to nine months, “depending on the treating orthopedic surgeon’s preference”.

Bias in the selection of the reported results corresponded closely to the “Selective reporting” domain for RCTs. The included NRSI was considered to be of “low risk” of bias in this domain.

Overall—The NRSI was assessed as “critical” due to a large amount of missing data and unclear selection of participants.

**Figure 2:**
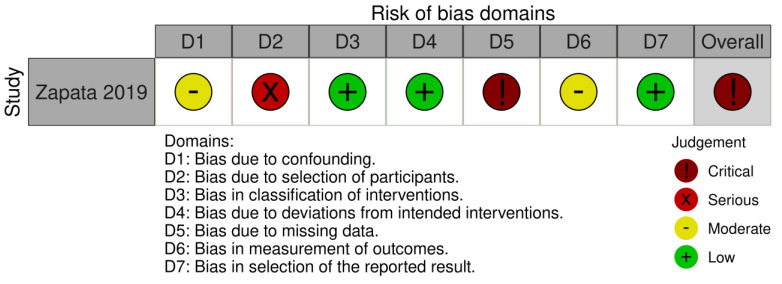
Risk Of Bias In Non-Randomized Studies - of Interventions (ROBINS-I) plot of the included non-randomized study of intervention (NSRI), with corresponding domains.

The ROBINS-I risk of bias plot is presented in Figure 2.

## 4. Discussion

Randomized controlled trials (RCTs) are considered the gold standard for researching therapeutic interventions because their design minimizes the likelihood of systematic error (bias), and as such they provide the strongest evidence to guide clinical decision making. Randomization protects against sampling bias, and any potential differences between the groups at baseline are deemed to occur due to chance. RCTs also guard against overenthusiastic conclusions, which is often the case with uncontrolled trials. However, RCTs assume meticulous methodological design, conduct, and reporting, and not merely the usage of randomized allocation. Deviation from the truth in results can stem from various sources of bias. To appraise the risk of bias, Cochrane Collaboration proposed the usage of the RoB 2 tool for appraisal of RCTs [52] and ROBINS-I for NRSIs [53]. These tools help to differentiate the type of systematic errors that can occur at the beginning, during, or in the reporting of trial results. Sources of bias can be broadly grouped into biases that stem from the randomization process, deviations from intended interventions, missing outcome data, measurement of the outcomes, and selection and reporting of the results. Knowledge syntheses aim to collate the evidence on a specific research question to provide valid information about the effectiveness of an intervention. Thus, rigor in RCT conduct and reporting is essential.

The CONSORT statement is a set of guidelines for reporting on RCTs [57,58,59]. It consists of a checklist of necessary information that should be included in RCT reporting and a flow diagram that documents the movement of participants from screening through reporting of the trial. The goal of CONSORT is to improve the transparency, completeness, and quality of RCT reporting. Most reputable medical journals have adopted CONSORT guidelines and require authors to use them.

Our analysis showed that overall, reporting of the Schroth PSSE trials was poor, and they currently lack methodological rigor.

In summary, seven studies met inclusion criteria for this review. Five studies reported to have used the Schroth method, and two the Barcelona-Scoliosis-Physical-Therapy-School approach. One study included patients with Scheuermann’s kyphosis [60] and six with adolescent idiopathic scoliosis [51,61,62,63,64,65]. All but one [64] were RCTs. The inclusion criteria varied among studies.

All studies included pediatric populations. Using a search term “scoliosis” in the clinicaltrials.gov database, we identified two registered clinical trials investigating the effect of the Schroth method in adults, one being conducted in Canada and another in Pakistan. However, our search did not yield any published RCTs or NRSIs including adults.

Studies included in this review were conducted in Turkey (*n* = 2), and one each in Canada, Israel, the Republic of Korea, Saudi Arabia, and the USA. Intervention periods were long-term for two studies (1-year follow-ups) and intermediate for five studies (range: 10 weeks to 6 months).

The Scoliosis Research Society (SRS) recommendation for standardizing non-operative research identifies the Cobb angle as the primary outcome [66]. Consequently, the primary outcome for all studies was the Cobb angle (measured via X-ray in all but one study, which used surface topography). Other outcomes included: quality of life, incidence of progression, brace prescription, angle of trunk rotation, static plantar pressure distribution, functional capacity, muscle endurance, waist asymmetry, weight distribution, pain, body image perception, surface topography measurements, and spinal mobility. The diversity in the outcome selection and varied inclusion criteria makes it difficult to compare trial results. A core set of outcome domains and measurements is critical for establishing the effectiveness of an intervention and comparison across similar trials. In a four-round Delphi study, The Nordic Spinal Deformity Society (NSDS), proposed 13 core outcomes for surgically treated children with AIS, which prioritizes patient-reported outcomes and lists only three clinical outcomes, including “re-operation”, “complications”, and “change in deformity” [67]. Our group recently conducted a scoping review to inventory the tools that measure patient-reported outcomes in persons with spinal deformities and identified 145 patient-reported outcome measures [68]. The creation of core outcome sets for non-operatively treated children and adults with scoliosis has gained global attention. However, consensus has not been reached. Currently, Close et al. are conducting a qualitative investigation to establish a person-centered core set for adolescents and young adults with spinal deformity who are undergoing treatment [69].

Adherence was explicitly monitored in three and exercise performance in two studies. Exercise progression was reported in two studies. Exercise prescription according to the Schroth curve type was reported in one trial. Stratifying randomization by curve type was reported in one study.

Four studies did not register their protocols, two registered after study completion, and one after the study commenced but before completion. Only one study provided sufficient information in the published protocol to support replication. Insufficient information for replication of the study or treatment protocols makes it difficult to judge the study’s conduct, validity, and the intervention outcomes.

One study was determined to be of appropriate quality for risk of bias, while others scored sub-optimally on multiple domains.

Authors typically do not report classifying participants into a specific Schroth classification. Several classifications for scoliosis exist to guide the selection of surgical procedure, and the most used among surgeons in North America is the Lenke classification developed in 2001 [70]. Lenke classification distinguishes between six types of scoliosis patterns, each of which can be subdivided according to the extent of lumbar deviation and sagittal plane profile. Classification systems help clinicians to reliably compare among various procedures and to assess outcomes in different curve types. While Lenke classification relies on the radiological characteristics of the curve, the Schroth classification is based on the clinical presentation, distinguishing between different compensatory patterns that guide therapy decisions. Without a Schroth classification, a Schroth therapist cannot reliably prescribe the exercise treatment. Thus, to ensure an appropriate Schroth therapy prescription, it is essential to use the Schroth classification.

Three studies reported using certified Schroth therapists. Not using qualified Schroth therapists challenges the trustworthiness of the intervention and, thus, the validity of the results. The Schroth method, among other PSSE methods, is a specialized physiotherapeutic treatment for scoliosis and hyperkyphosis that requires additional postgraduate training beyond what a typical physical therapy curriculum offers. Schroth therapists undergo an advanced theoretical and clinical postgraduate education to establish a broader depth of knowledge and skills related to the treatment of patients with scoliosis and hyperkyphosis. The certification process requires them to demonstrate competency in specialized knowledge and advanced clinical proficiency on the topic. Moreover, the validity of knowledge syntheses depends on reliable intervention administration. Administering medication in pharmaceutical trials is far less complex than delivering a specialized physiotherapeutic intervention. Thus, it is critical that Schroth therapists participate in the studies examining the effect of Schroth intervention.

A limitation of this review is the inclusion of only Schroth PSSE trials. The Schroth method was specifically developed to treat persons with adolescent idiopathic scoliosis and was later extended to treat patients with hyperkyphosis and adult spine deformity. To date, it remains the most researched method, despite the existence of other exercise methods for scoliosis. Hence, in this study, we focused on Schroth trials. However, our recommendations could be extrapolated to other physiotherapeutic trials, and particularly to those that examine the effect of the PSSE more broadly. The evidence synthesis depends on the quality of the primary studies, and high standards in trial design and conduct are imperative.

## 5. Conclusions

We recommend minimum standards for the reporting of Schroth PSSE trials, including prospectively registering detailed study protocols, providing appropriate PSSE labeling, utilizing the Schroth classification system and qualified Schroth therapists, naming and describing specific exercises per Schroth curve type, and providing therapy dosages, prescription methods, and adherence. This will strengthen the evidence base for Schroth PSSE and ensure that collation of evidence through systematic reviews is valid.

## Data Availability

No new data were created or analyzed in this study. Data sharing is not applicable to this article. However, all pertaining information to this review is available in the Supplementary Materials or is contained within the article.

## Supplementary Materials

The following supporting information can be downloaded at: https://www.mdpi.com/article/10.3390/children10060954/s1, File S1: Search strategy; File S2: Domains of interest; File S3: Risk of bias and study quality assessment; File S4: Reported variables across included studies.
