# Supplementary material for: Schroth Physiotherapeutic Scoliosis-Specific Exercise (PSSE) Trials—Systematic Review of Methods and Recommendations for Future Research
Source: Children (Basel). 2023 May 27;10(6):954. doi: 10.3390/children10060954 (PMC10297476; doi:10.3390/children10060954)
Supplement: Supplementary file 1 [file children-10-00954-s001.zip › children-2219861-supplementary.pdf]

Supplementary File S1

Search Strategy

- PubMed: schroth*[tw]
- Embase: schroth*:ti,ab,kw
- Scopus: TITLE-ABS-KEY (schroth*) --> use "Advanced Document Search" link (under search box)
- CINAHL: TI schroth* OR AB schroth*
- SPORTDiscus: TI schroth* OR AB schroth*
- PEDro: schroth*

Supplementary File S2

Domains of Interest Extracted for the Systematic Review

General Characteristics	Treatment Protocols
1. Study ID<br>2. Title<br>3. Lead author's Name<br>4. Lead author's affiliation<br>5. Lead author's contact<br>6. Country in which the study was conducted<br>7. Setting<br>8. Study funding sources<br>9. Possible conflicts of interest for study authors	1. Name of the Schroth exercises<br>2. Were the participants treated by a certified Schroth therapist?<br>3. How many Schroth therapists were involved in the study?<br>4. Intervention description other than the Schroth<br>5. Start date<br>6. End date<br>7. Duration of study period<br>8. Intensity of the Schroth treatment<br>9. How was the intensity of the Schroth intervention progressed?<br>10. How was adherence monitored<br>11. How was adherence calculated?<br>12. How was performance monitored
**Methodological Approaches**<br>1. Aim of study<br>2. Study design<br>3. Population description<br>4. Description of the curve types<br>5. Inclusion criteria<br>6. Exclusion criteria<br>7. Method of recruitment of participants<br>8. Was the study protocol registered in an online registry of trials?<br>9. Did the authors report to have classified the patients according to their curve type?<br>10. If the authors reported to have classified the patients according to the curve type, what classification system did they report using?<br>11. Was bracing used in study participants?<br>12. Were the Schroth exercises used as an intervention or control?<br>13. Outcomes under investigation<br>14. Questionnaires used to assess PROMs	**Outcomes Reporting**<br>1. Total number of participants<br>2. Baseline Population Characteristics:<br>3. Age Schroth<br>4. Age Other intervention<br>5. Age p-value<br>6. Sex Schroth<br>7. Sex Other intervention<br>8. Sex p-value<br>9. Ethnicity/Race Schroth<br>10. Ethnicity/Race Other intervention<br>11. Ethnicity/Race p-value<br>12. Risser sign Schroth<br>13. Risser sign Other intervention<br>14. Risser sign p-value<br>15. Classification Schroth<br>16. Classification Other intervention<br>17. Classification p-value<br>18. Cobb angle Schroth<br>19. Cobb angle Other intervention<br>20. Cobb angle p-value

Supplementary File S3

Risk of Bias (RoB) and Study Quality Assessment

(a) Augmented RoB 2 tool designed for the methods review

D1 - Sequence generation
Describe the method used to generate the allocation sequence in sufficient detail to allow an assessment of whether it should produce comparable groups.

D2 - Allocation concealment
Describe the method used to conceal the allocation sequence in sufficient detail to determine whether intervention allocations could have been foreseen in advance of, or during, enrolment.

D3 - Blinding of participants
Describe all measures used, if any, to blind study participants from knowledge of which intervention a participant received. Provide any information relating to whether the intended blinding was effective.

D4 - Blinding of the study therapists
Describe all measures used, if any, to blind study therapists from knowledge of which intervention a participant received. Provide any information relating to whether the intended blinding was effective.

D5 - Blinding of outcome assessment - evaluator
Describe all measures used, if any, to blind outcome assessors from knowledge of which intervention a participant received. Provide any information relating to whether the intended blinding was effective.

D6 - Blinding of outcome assessment - statistician
Describe all measures used, if any, to blind statistician from knowledge of which intervention a participant received. Provide any information relating to whether the intended blinding was effective.

D7 - Incomplete outcome data
Describe the completeness of outcome data for each main outcome, including attrition and exclusions from the analysis. State whether attrition and exclusions were reported, the numbers in each intervention group (compared with total randomized participants), reasons for attrition/exclusions where reported, and any re-inclusions in analyses performed by the review authors.

D8 - Attrition
Was dropout rate described and acceptable. In a short-term study, a drop-out of >20% is unacceptable. Likewise, in a long-term study, a drop-out of >30% in unacceptable.

D9 - Selective reporting
State how the possibility of selective outcome reporting was examined by the review authors, and what was found. Compare the published protocol if available to the publication report.

D10 - Baseline group characteristics
Were the study groups similar at baseline regarding the demographics and, especially main outcome of the study?

D11 - Cointervention
Were the cointerventions reported, were they similar among groups, did the investigators tried to minimize cointervention, was the study free of cointervention? If patients received bracing, this is not to be considered cointervention as bracing is standard of care. Consider high risk of bias if this was not controlled for and specified a priori.

D12 - Compliance
Was the compliance monitored, if so how, was it acceptable based on the treatment prescription?

D13 - Outcome detection
Was the timing of the outcome assessment similar in all groups?

D14 - Other sources of bias (a "catch-all" domain)
State any important concerns about bias not addressed in the other domains in the tool. If questions/entries were pre-specified in the review's protocol, responses should be provided for each question/entry.

(b) Risk Of Bias In Non-Randomized Studies - of Interventions (ROBINS-I) tool

RoB 2 and ROBINS–I are mostly distinct in the first 3 domains, whereas the D4, 5, 6, 7 and 8 correspond closely to RoB 2. The first two domains could be affected at the pre-intervention phase, the last three at the post-intervention, and bias in classification of intervention occurs during the intervention.

D1 - Bias due to confounding
Were the baseline characteristics comparable? Did the authors include participants with one or more predictors that would predict the outcome? For example, in the context of the Schroth trials, more mature patients in the control group would mean less risk of progression and would likely predict better outcomes in the exercise group. Other potential confounders include previous exposure to exercise intervention, curve magnitude, bracing, comorbidities, etc. Did the authors control for any post-intervention variables that could have been affected by the intervention?

D2 - Bias due to selection of participants
How was the sample generated? Did the authors include a convenience sample, those who already participated and were knowledgeable of the treatment, new participants? Was selection of participants into the study (or into the analysis) based on participant characteristics observed after the start of intervention? Do start of follow-up and start of intervention coincide for most participants?

D3 - Bias in classification of interventions
Is there a risk of incorrect group assignment? Were the intervention groups clearly defined? Was the information used to define intervention groups recorded at the start of the intervention? Could classification of intervention status have been affected by knowledge of the outcome?

D4 - Bias due to deviations from intended interventions
Were co-interventions reported and balanced across groups? Was the intervention implemented successfully for most participants? Did study participants receive the intervention as allocated?

D5 - Bias due to missing data
Corresponds closely to "Attrition" of RoB 2 tool.
Was dropout rate described and acceptable? In a short-term study, a drop-out of >20% is unacceptable. Likewise, in a long-term study, a drop-out of >30% in unacceptable. Was the dataset reasonably complete? Were some data excluded from the analysis and reasons for exclusion?

D6 - Bias in measurement of outcomes
Corresponds closely to "Outcome detection" of RoB 2 tool.
Was the timing of the outcome assessment similar in all groups? Could the outcome measure have been influenced by knowledge of the intervention received? Were the assessors blinded?

D7 - Bias in selection of the reported result
Corresponds closely to "Selective reporting" domain of RoB 2 tool.
State how the possibility of selective outcome reporting was examined by the review authors, and what was found. Compare the published protocol if available to the publication report. Were multiple analyses done for an outcome but only a subset reported?

D9 – Overall (a "catch-all" domain)
State any important concerns about bias not addressed in the other domains in the tool. If questions/entries were pre-specified in the review's protocol, responses should be provided for each question/entry.

Supplementary File S4

Reported Variables Across Included Studies

	Age		Sex		Race/Ethnicity*		Risser score		Mean Cobb angle (°)		Classification	
**Study**	**Schroth**	**Control**	**Schroth**	**Control**	**Schroth**	**Control**	**Schroth**	**Control**	**Schroth**	**Control**	**Schroth**	**Control**
**Randomized controlled trials (RCTs)**												
**Kim 2016** (n=24)	15.60±1.1	15.60±1.1	All F	All F	NR	NR	NR	NR	23.6±1.5	24.0±2.6	NR	NR
**Kuru 2016** (n=45)	Supervised 12.9; Non-supervised 13.1	12.8	Supervised 14F, 1M; Non-supervised 12F, 3M	13F,2M	NR	NR	1.5±1.3	1.0±1.2	33.4±8.9	30.3±6.6	NR	NR
**Schreiber 2016** (n=50)	13.5 (95% CI 12.7, 14.2)	13.3 (95% CI 12.7, 13.9)	23 F	24 F	NR	NR	1.76	1.44	Largest curve: 29.1 (95%CI 25.4, 32.8) Sum of curves: 48.1 (95%CI 39.1, 57.2)	Largest curve: 27.9 (95%CI 24.3, 31.5) Sum of curves: 54.3 (95%CI 44.9, 63.6)	3c (n= 7) 3cp (n= 15) 4c (n= 5) 4cp (n= 23)	
**Bezalel 2019** (n=50)	14.52±1.79	13.39±1.66	20% M	20% M	NR	NR	2 (1-4)	2 (0.25-4)	60.18±8.38	56.16 ±7.59	Scheuermann's kyphosis	Scheuermann's kyphosis
**Kocaman 2021** (n=28)	14.07±2.37	14.21±2.19	10 F, 4 M	11 F, 3 M	NR	NR	1.64±1.34	1.78±1.19	Thoracic:17.64±4.01 Lumbar: 15.80±3.42	Thoracic: 17.29±3.45 Lumbar: 15.17±4.02	R thoracic: 3 L thoracic: 5 R thoracic/ L lumbar: 6	R thoracic: 3 L thoracic: 5 R thoracic/ L lumbar: 6
**Mohamed 2021** (n=34)	14.50 ± 1.20	14.90±1.40	17 F	17 F	NR	NR	Risser II: 6 Risser III: 5 Risser IV: 6	Risser II: 5 Risser III: 5 Risser IV: 7	20.42 ± 2.57	20.21 ± 2.80	17 TL	17 TL
**Non-randomized studies of intervention**												
**Zapata 2019** (n=33)	12.5±1.5	11.8±0.9	12 F, 7 M	14 F	Caucasian:16 Hispanic:2 Asian: 1	Caucasian:11 Hispanic:1 Asian: 1	Risser 0: all Open TRR: 16 Closed TRR: 3	Risser 0: all Open TRR: 12 Closed TRR: 2	16.3±3.4	16.0±3.2	Thoracic: 3 Double major: 6 TL/L: 10	Thoracic: 5 Double major: 4 TL/L: 5

Abbreviations:
NR – not reported
Sex: F – female; M – male
Classification: R – right; L – left; TL – thoracolumbar; TL/L – thoracolumbar/lumbar

*Race/ethnicity categories labelled according to how reported in the cited paper
